# Community Interactions Modify the Effects of Pharmaceutical Exposure: A Microcosm Study on Responses to Propranolol in Baltic Sea Coastal Organisms

**DOI:** 10.1371/journal.pone.0093774

**Published:** 2014-04-08

**Authors:** Hanna Oskarsson, Ann-Kristin Eriksson Wiklund, Gunnar Thorsén, Gabriela Danielsson, Linda Kumblad

**Affiliations:** 1 Department of Ecology, Environment and Plant Sciences, Stockholm University, Stockholm, Sweden; 2 Department of Applied Environmental Science, Stockholm University, Stockholm, Sweden; 3 Department of Analytical Chemistry, Stockholm University, Stockholm, Sweden; 4 Department of Biochemistry and Biophysics, Stockholm University, Stockholm, Sweden; University of Auckland, New Zealand

## Abstract

This study investigated the uptake and effects of a common human pharmaceutical, propranolol, on the structure and function of a coastal Baltic Sea model community consisting of macroalga (*Ceramium tenuicorne*), mussels (*Mytilus edulis trossulus*), amphipods (*Gammarus* spp.), water and sediment. The most sensitive species, the mussel, was affected to the same extent as in previous single species studies, while the effects on the amphipod and alga were smaller or even positive compared to experiments performed in less complex test systems. The observed cascade of beneficial effects was a result of inter-specific species interactions that buffered for more severe effects. The poor condition of the mussel led to a feeding shift from alga to mussel by the amphipods. The better food quality, due to the dietary shift, counteracted the effects of the exposure. Less amphipod grazing, together with increased levels of nutrients in the water was favourable for the alga, despite the negative effects of propranolol. This microcosm study showed effects on organisms on different organizational levels as well as interactions among the different components resulting in indirect exposure effects of both functional and structural nature. The combination of both direct and indirect effects would not have been detected using simpler single- or even two-species study designs. The observed structural changes would in the natural environment have a long-term influence on ecosystem function, especially in a low-biodiversity ecosystem like the Baltic Sea.

## Introduction

Our knowledge on the potential effects of pharmaceuticals on non-target organisms has increased over the last years, yet there is a lack of studies on the effects of pharmaceuticals on ecosystem structure and function, and on ecosystem processes [1]. In complex systems with variations within species as well as inter-specific species interactions, exposure to environmental stressors can result in various indirect and cascading effects, with positive or negative consequences on individual species and communities [2], [3]. Differences between species can lead to unexpected effects in response to exposure [4], and compensatory processes in ecological systems can result in both linear and non-linear linkages between effects on individuals and subsequent effects on populations [5]. Ecological buffering capacity and other aspects of ecosystem dynamics and stability can furthermore shield the effects of stressors on an ecosystem [6], resulting in less distinct direct effects than can be detected for species or individuals. This can for instance occur if several species benefit from the same resources, that is, fulfil the same functional roles with the ability to replace one another’s function in the system, or as a result of positive indirect effects within the system [6]. Single species experiments could therefore both over- and underestimate the hazards of exposure to toxic substances, and ecotoxicological measures on population level should therefore better predict the effects on ecosystems than measures on individual level [7]. Theories of ecosystem responses to disturbances, unexpected future ecosystem events and the importance of observing change on larger scales derive from the concept of ecosystem resilience, introduced by Holling [8] and reviewed by for example Rönnbäck et al. [9] for the Baltic Sea context. With this in mind, multi-species exposure studies of function and structure are important complements to single-species studies on mechanistic responses, to better understand the possible effects of pharmaceuticals and other environmental contaminants on organisms and ecosystems.

The Baltic Sea is subjected to considerable natural and anthropogenic stress [10], and is considered to be one of the most polluted seas in the world [11]. There is a strong salinity gradient, thermo- and halo-clines and irregular inflows of oxygen rich water from the Atlantic [12]. This contributes to a low biodiversity in the Baltic Sea ecosystem, where each species is of high importance, as the functional redundancy is low [9], [13]. Coastal ecosystems function as buffers for nutrient flows from the terrestrial to the open sea ecosystems. In the Baltic Sea, the blue mussels, *Mytilus edulis trossulus*, together with perennial algae, have essential stabilising functions in this process [14], [15].

Pharmaceuticals constitute an important group of environmental contaminants due to their biologically active nature, the conservation of drug targets in non-target organisms and their continuous release into the environment via waste water treatment plants. Pharmaceuticals are often relatively water soluble, designed to be effectively absorbed, to induce biological effects [4] and to be biologically persistent [16]. Consequently, this diverse group of substances can pose risks of both bioaccumulation and effects in non-target organisms [17]. Propranolol is a common β-blocker primarily used for treating of hypertension [4]. Propranolol is often detected in aquatic environments, and in the approximate range of 0.01–0.1 μg l^−1^ in the Baltic Sea catchment area [18]–[21]. Being a non-specific β-blocker, propranolol binds to both β1- and β2-receptors in humans [22], which in turn inhibit the action of catecholamines [23]. β-receptors have been shown to be present in tissues of fish [24], and possibly also in bivalves [25]–[27] as well as in a range of other organisms [24]. It is hence possible that propranolol exert a similar mode of action as in humans, also in non-target organisms. Previous studies on the effects of propranolol on aquatic organisms have shown effects within a large range of exposure concentrations, dependent on both exposure durations, test species and endpoints. Tests with for example algae, crustaceans and fish have revealed EC50’s ranging from 0.5–24300 μg l^−1^ [28]–[30], and NOEC’s for small crustaceans from 1–125 μg l^−1^ [24]. Previous single species experiments have shown effects of propranolol also in aquatic organisms from coastal habitats in the Baltic Sea, in concentrations ranging from 10–10000 μg l^−1^ [31]–[33].

To study the direct, indirect and possible buffering effects in a community exposed to pharmaceuticals, we performed a laboratory multiple-species microcosm study. We studied the effects of the common human pharmaceutical propranolol on a model community including organisms with different feeding modes and from different biological organisational levels. The setup allowed studies of the propranolol distribution within the community, and served as a comparison to our previous experiments, which showed effects of propranolol, diclofenac and ibuprofen in less complex experimental systems with one and two species [31]–[33]. By mimicking a coastal Baltic Sea community, we hypothesised that we would find smaller negative effects of exposure than in less complex systems. Based on the detected interactions and indirect effects within the model community, the study provides novel insights into how pharmaceuticals can affect food web structure and ecosystem functions in the field.

## Material and Methods

### 2.1 Overview of the Experimental Set up

In this study, model communities (containing macroalga, omnivorous amphipods, filter feeding mussels, water and sediment) were created in aquaria connected to a flow through system with fresh seawater. Three treatments (control and exposure to two propranolol concentrations) were studied in five replicates (n = 5) during six weeks in a climate chamber laboratory. At the start, during and after the exposure experiment, measurements were conducted to identify potential effects of the propranolol exposure.

### 2.2 Study Organisms

The model communities studied in this experiment were formed to resemble a habitat of a coastal ecosystem, with natural abundances of the respective organisms at the sampling site [34]–[36]. Each community was represented by: i) blue mussels (*Mytilus edulis trossulus,* 31 individuals of even size, 2.3 cm±se 0.006, cleaned from epibionts), ii) a red filamentous macroalgae (*Ceramium tenuicorne,* initial mean weight 8.3 g ww±se 0.01), iii) small amphipods (*Gammarus* spp., 30 evenly sized individuals, appr. 1–1.5 cm), as well as iv) sediment (in petri-dishes Ø 10*1.5 cm) and v) water.

Sampling of organisms and sediment was performed by diving, by hand and by a sediment sledge (1–3 m depth), the 20^th^–21^st^ of June 2011 in the vicinity of Stockholm University’s Baltic Sea Center, Askö Laboratory (58°49′3′′ N, 17°37′4′′ E) in the Baltic Sea, Sweden. The salinity and water temperature at sampling was 6.5 psu and 13.4°C, respectively. The sediment was sieved through a 3 mm net to remove larger organisms and larger inorganic material.

No permits were required for the described study, which complied with all relevant regulations. No protected species were sampled during the collection of organisms and sediment.

### 2.3 Experimental Conditions

The sampled organisms and sediment were distributed among 15 glass aquaria (LWH 24*19*18 cm, volume 8 l) and acclimated for seven days in a climate chamber at Stockholm University, with a water temperature of 12.5±0.5°C and the light regime 16∶8 h (light:dark, 11.11±se 0.72 UM (μmol s^−1 ^m^−1^)) before exposure. The same conditions persisted throughout the experimental period. Fresh seawater (salinity 6.52±se 0.0075 psu) was continuously added into each separate aquaria via a flow through system, releasing the water through PVC tubings (inner Ø 8 mm) at approximately 5 cm from the bottom. The daily incoming water corresponded to the aquaria volume, so that the total water volume of each aquarium was exchanged daily. Surplus water was continuously discharged through a hole in the aquaria glass wall at the water surface. The hole was covered with a fine net to avoid escape by amphipods or losses of alga.

Salinity and temperature of the water were measured regularly. A daily addition of 10 ml solution of the microalgae *Isochrysis galbana* (Reed Mariculture) to each aquarium ensured sufficient food supply for the mussels. The microalgae concentration in the aquaria after addition was 7.8×10^7^ cells l^−1^, or 2–3 mg dw l^−1^, according to relationships from Lora-Vilchis et al. [37] and Zhue and Lee [38] and measured dry weight of the microalgae. The administered amount (in cells l^−1^, cells l^−1^ mussel^−1^ or mg dw l^−1^) was similar to several previous studies [31], [39]–[41].

### 2.4 Exposure

The model communities were exposed to 0 (control), 100 (P100) and 1000 (P1000) μg l^−1^ of propranolol (nominal concentrations), n = 5. These concentrations have been previously used in single and two-species exposure studies with propranolol [32], [33], [42]. The propranolol, purchased from Sigma Aldrich, was dissolved in dilute phosphoric acid and the pH was adjusted to 7.1 by the addition of a buffer (bisodium carbonate). This buffer was also added to the control treatment to control for solvent effects, although previous studies showed no difference between solvent control and seawater controls [32]. Propranolol and buffer were continuously added to the aquaria by siphons of PTFE Teflon tubing (length: 30 cm, inner Ø 0.5 mm) connected to fused silica capillaries (length: 15 cm, inner Ø 0.25 mm). The siphons led propranolol or buffer from glass reservoirs placed on shelves above the aquaria, into the water of each experimental aquaria. The aquaria were distributed randomly in the climate chamber to minimize possible effects from placement in the room.

### 2.5 Measurements of the Model Communities and its Separate Components

#### 2.5.1 Nutrients in water

Water samples from all aquaria were collected at the end of the exposure period and kept cool (4°C) and dark until analysis of ammonium (NH_4_^+^), nitrite+nitrate (NO_2_^−^+NO_3_^−^), total amount of nitrogen (Tot-N) and phosphate (PO_4_^3−^). The nutrients were determined by Segmented Flow Analysis (SFA), slightly modified ALPKEM O. I. Analytical Flow Solution IV Methods (#3199527, NO_2_+NO_3_ – N; #319526, NH_4_ – N and #319528, PO_4_ – P).

#### 2.5.2 Propranolol in water

Quantification of propranolol in water samples from two selected aquaria of each concentration (0, 100, 1000 μg l^−1^) was performed twice during the exposure period (day 2 and day 15), and from all aquaria at the end of the exposure, to assess that the concentrations in the aquaria remained constant. Water samples were collected in glass vials and kept cool and dark until analysed according to the method described by Eriksson Wiklund et al. [32].

#### 2.5.3 Propranolol in biota and sediment

Quantification of propranolol in mussels, amphipods and sediment samples from selected aquaria was performed at the end of the exposure period. Analyses were made for 1–3 mussels from three different aquaria per exposed treatment (100 μg l^−1^ and 1000 μg l^−1^) and for one control aquaria (0 μg l^−1^); for 1–2 amphipods per treatment (analysed together) and for one sub-sample of sediment from 2–3 aquaria per treatment. The mean±se for mussels in P1000 was based only on data from two of the analysed aquaria. The third replicate (one individual showed a considerably higher propranolol concentration than the other analysed individuals from P1000. It was identified as an outlier (Dixon test, p = 0.02) and excluded from the overall mean. Biota and sediment samples were dried (45°C 3 days) and kept cool and dark until analysed. Macroalgae samples could not be analysed due to laboratory circumstances.

The propranolol content in blue mussels was analysed according to the earlier developed methods [30], [31] with some minor changes. The instrument used was a Shimadzu gradient liquid chromatography system (LC-10Avp) coupled to a Thermo Scientific ion trap mass spectrometer (LCQ-deca classic).

The following sample preparation steps were used for the mussel tissue and sediment: rewetting and homogenisation of the tissue, addition of surrogate standard (d_7_-propranolol, CDN isotopes, Pointe Claire, CAN, product no: D-2386), extraction for more than 3 hours using formic acid (2%) in methanol with 30 minutes ultrasonication, centrifugation to remove solids, buffer exchange to ammonium -acetate buffer (0.1%) containing 20% acetonitrile (ACN), liquid-liquid extraction with n-heptane to remove fat components, solid phase extraction of basic compounds using Oasis MCX columns (Waters, Millford, MA, USA), evaporation of eluate and reconstitution in ammonium-acetate buffer (0.1%) containing 20% acetonitrile. The mussels were analysed individually and approximately 1 g (dry weight) of each sediment sample was weighed on an analytical scale.

Amphipod samples were frozen in liquid nitrogen and ground to a fine powder. Surrogate standard was added after transfer of the powder to Eppendorf tubes for extraction. The extraction was performed as for the mussel samples. Centrifugation was used to remove particulate material and 30 kD molecular weight cut-off filters to remove proteins (Ultracel YM-30, Millipore, Bedford, MA, USA). The solvent was evaporated and the residue dissolved in ammonium-acetate buffer (0.1%) containing 20% acetonitrile.

The injection volumes used for the LC-MS analysis depended on the expected levels in the sample, 1 μl for high concentration samples and 5 μl for low concentration samples. Two fragments were monitored from the ions of the non-labeled and deuterium labeled propranolol in order to assess that the measured signals originated from the desired compound: the fragments at m/z = 157 and 183 were monitored for the parent ion 260 and the fragments at m/z = 163 and 189 for the parent ion 167. All chemicals used were of analytical grade and all water used was double deionised using a Milli-Q water purification apparatus (Millipore, Bedford, MA, USA), having a resistance greater than 18 MΩ.

Bioconcentration factors (BCFs) for the biota and concentration factors (CFs) for the sediment samples were determined as the ratio between detected amounts of propranolol in biota or sediment, and detected concentrations of propranolol in the water of the respective aquaria. The relative distribution of propranolol in the microcosms was estimated from the measured concentrations in the respective microcosm components (assuming a similar concentration in the alga and the amphipod) and the initial (bio)mass of each component.

#### 2.5.4 Community Gross Production (GP) to respiration (R) Ratio - GP∶R

The gross production to respiration ratio (GP∶R) of the model communities was measured three times; after the acclimatisation period but before the start of the exposure, as well as after 4.5 weeks of exposure and at the end (6 weeks of exposure). Oxygen measurements were conducted with a microsensor connected to a pA-meter (Unisense). After measuring the initial oxygen concentration (t_0_), the water surfaces of the aquaria were covered with plastic film to limit oxygen diffusion over the water surface, and the systems were subsequently left in light conditions for a known period of time before the oxygen concentration was measured again (t_1_), to attain the net primary production (NP). The water surfaces were subsequently re-covered with plastic film and the systems were left in dark conditions, before the oxygen concentration was measured a third time (t_2_), to attain the respiration (R) of the systems. Potential changes in oxygen level not due to organism production and/or respiration were controlled for using blanks containing only seawater. The gross production (GP) was estimated as the sum of NP and R. All production and respiration measurements were related to the water volume of the aquaria.

#### 2.5.5 Measurements of macroalgae

Gross production to respiration ratio (GP∶R) of the macroalgae was measured twice, after 5 weeks of exposure and at the end. The measurements were performed in the same manner as for the model communities. During the measurements the macroalgae were removed from the aquaria and kept in 1 l plastic jars with seawater and the respective concentration of propranolol or buffer. After the measurements after 5 weeks of exposure, the algae were replaced in their respective aquaria again. The measurements were normalised to water volume of the aquaria and alga biomass (dw). Dry weight of the macroalgae after 5 weeks was attained from wet weights, normalised by the relation between dry weight and wet weight at the end of the experiment.

#### 2.5.6 Measurements of mussels

The mussel respiration rate was measured only at the end of the exposure period to avoid extra stress from detachment of byssus threads from their substrate. The measurement was performed in the same way as the respiration measurements described for the GP∶R measurements for the whole system. During the measurements all mussels were removed from the aquaria and kept in 1 l plastic jars with seawater and the respective propranolol concentration or buffer. Directly after the respiration measurement the mussel feeding rate was also measured. The feeding rate measurements were performed in the same jars, and started with the addition of microalgae solution (1 ml) to each jar, resulting in a concentration of 3.9×10^7^ cells l^−1^ and 0.95 mg dw l^−1^. Initial water samples (50 ml, t_0_) were taken with a 60 ml syringe, and transferred into 50 ml Falcon tubes. The water was gently stirred while the water samples were taken to obtain an as homogenous sub-sample as possible. The mussels were then left to filter feed for 21±1 min, before the water was sub-sampled again (t_1_), in the same way. The water samples were stored cold and dark until analysed for number of cells 3–5 μm in a particle counter (Beckman Coulter Z1 DT). The respiration and feeding rate measurements were normalised to water volume and biomass (dw).

#### 2.5.7 Measurements of amphipods

The amphipod respiration was also measured at the end of the exposure period and was estimated from the measurement of two individuals from each aquarium. The amphipods were transferred to two 25 ml plastic jars with seawater and the respective concentration of propranolol or buffer. The measurements were conducted in the same way as for the respiration measurements described for the GP∶R measurements, and were normalised to water volume and biomass (dw).

#### 2.5.8 Measurements of sediment

Measurements of gross production, respiration, and GP∶R, of the sediment were performed at the end of the experiment, in the same way as for the model communities. During the measurements the sediment samples (in their respective Petri dishes) were removed from the aquaria and kept in 0.5 l plastic jars with seawater and the respective concentration of propranolol or buffer. The measurements were normalised to water volume and sediment mass (dw). Dry weight of the sediment was attained from initial wet weights, normalised by the relation between dry weight and wet weight of a subsample at the end of the experiment.

#### 2.5.9 Mortality

The communities were controlled daily to identify possible dead mussels and/or amphipods. Dead organisms were removed and stored in −20°C for later measurements of length and weight.

#### 2.5.10 Dry weight, ash free dry weight and carbon content

The dry weight and ash free dry weight of mussels, amphipods, macroalgae and sediment was attained after the completed exposure. Mussel soft tissue was removed from the shell. All samples were placed in pre-ashed (450°C, 5 h) aluminium cups, and then left to dry (45°C, 3 days) before weighing. Thereafter the samples were ashed (500°C, 2 h) in the same aluminium cups in a muffle furnace before weighed again. The carbon content (%) was determined as the difference in weight between dry samples and ashed samples, divided by dry weight.

#### 2.5.11 Visual observations

Observations of possible changes among the communities were made throughout the exposure, and in detail at the end. Colour, structure and abundance of the macroalga, as well as bacteria/epiphytes on the aquaria walls were recorded.

### 2.6 Statistical Analyses

To determine differences in measured variables between the three treatments, continuous data was analysed in the statistical software package PRIMER 6 version 6.1.13, with the PERMANOVA+ add-on, version 1.0.3 [43], [44], specified for Euclidean distances and 9999 permutations. Prior to PERMANOVA analyses, homoscedasticity was tested by PERMDISP, in the PERMANOVA platform, and transformed values were used to reach homogenous variances if these were insufficient. Differences between treatments were determined by pair-wise PERMANOVA tests with Monte Carlo sampling as the number of unique permutations were low. Categorical data was analysed by generalized linear models (glm), specified for Poisson distribution, and significant effects were analysed with subsequent Tukey HSD post hoc tests, in R version 3.0.1. Correlation analyses were performed using Pearson correlation with logged data to reach homogenous variances (based on Levenes test for homoscedasticity) and pairwise removal of missing data, in SPSS version 20. All data is reported as mean±se if not otherwise stated.

## Results

### 3.1 Effects on the Model Communities

#### 3.1.1 Gross Production to respiration Ratio (GP∶R) in the model communities

The ecological function in each model community, assessed as community gross production to respiration ratio (GP∶R), was similar in all treatments before the start of the exposure (PERMANOVA: F_2,12_ = 0.089, p = 0.91), but different at the end of the exposure (PERMANOVA: F_2,12_ = 4.7, p = 0.024, Table 1). After the exposure, GP∶R was lower in communities exposed to 1000 μg l^−1^ (P1000) compared to the control (67% lower than control, pair-wise PERMANOVA: p = 0.047) and compared to communities exposed to 100 μg l^−1^ (P100), although not significantly (64% lower than P100, pair-wise PERMANOVA: p = 0.075). At the measurement after 4.5 weeks, GP∶R of the community was considerably affected, displaying negative values, but due to large variations, no statistical difference could be determined (PERMANOVA: F_2,12_ = 0.718, p = 0.51, Table 1).

**Table 1:** Results from nutrient analyses and physiological responses of the different components of the model communities.

Component	Variable (time)	Unit	Control	P100	P1000
**Community**	GP∶R (start)	mg l^−1^ h^−1^ g dw^−1^/mg l^−1^ h^−1^ g dw^−1^	1.57±0.045	1.53±0.071	1.52±0.119
	GP∶R (4.5w)	mg l^−1^ h^−1^g dw^−1^/mg l^−1^ h^−1^ gdw^−1^	0.661±0.169	0.701±0.359	−0.398±0.780
	GP∶R (6w)	mg l^−1^ h^−1^ g dw^−1^/mg l^−1^ h^−1^ g dw^−1^	1.42±0.062	1.31±0.115	0.469±0.394 *
	NO_2_^−^ +NO_3_^−^ (6w)	μg l^−1^	17.4±4.14	21.18±4.48	15.09±2.01
	NH_4_^+^ (6w)	μg l^−1^	140±15.8	141±25.1	317±75.7
	Tot-N (6w)	μg l^−1^	452±20	488±34	763±69 **
	PO_4_^3−^ (6w)	μg l^−1^	15048±1051	13132±643	14615±668
**Macroalga**	GP∶R (5w)	mg l^−1^ h^−1^g dw^−1^/mg l^−1^ h^−1^ g dw^−1^	1.44±0.217	1.43±0.335	0.93±0.463
	GP∶R (6w)	mg l^−1^ h^−1^g dw^−1^/mg l^−1^ h^−1^ g dw^−1^	1.52±0.271	2.12±.724	1.19±.463
	Weight loss (6w)	g ww	3.82±0.74	3.99±0.69	2.30±0.30
	Carbon content (6w)	g C g dw^−1^	0.76±0.005	0.79±0.009	0.83±0.007***
**Mussel**	Respiration (6w)	mg l^−1^ h^−1^g dw^−1^/mg l^−1^ h^−1^ g dw^−1^	−0.515±0.022	−0.549±0.037	−0.690±0.069
	Feeding rate (6w)	cells h^−1 ^g dw^−1^	13179±3920	19177±4358	18148±7089
	Weight (6w)	g dw	0.04±0.001	0.039±0.001	0.038±0.001
	Carbon content (6w)	g C g dw^−1^	0.880±0.004	0.876±0.004	0.885±0.005
	Mortality (6w)	amount (%) dead	1.3±0.8	3.2±1.0	54±11***
**Amphipod**	Respiration (6w)	mg l^−1^ h^−1^g dw^−1^/mg l^−1^ h^−1^ g dw^−1^	−2.68±0.31	−3.72±0.41	−2.51±0.28
	Juveniles (6w)	No.	9±4.9	13.4±6.1	25.2±9.7
	Juveniles/Adult (6w)	No.	1.5±0.9	0.81±0.3	1.90±0.9
	Weight (6w)	g dw	0.0074±0.0006	0.0075±0.0009	0.0085±0.004
	Carbon content (6w)	g C g dw^−1^	0.680±0.005	0.706±0.05	0.752±0.051
	Mortality (6w)	amount (%) dead	77±6.1	64±12	51±9.6***
**Sediment**	GP∶R (6w)	mg l^−1^ h^−1^g dw^−1^/mg l^−1^ h^−1^ g dw^−1^	1.17±0.15	0.94±0.14	0.95±0.0003
	Carbon content (6w)	g C g dw^−1^	0.0249±0.0008	0.0254±0.0006	0.0248±0.0006

Measurements made at start (start), after five weeks (4.5w and 5w), and/or after six weeks (6w) exposure to propranolol in 0 (Control); 100 μg l^−1^ (P100) and 1000 μg l^−1^ (P1000), mean ± se. dw = dry weight, ww = wet weight, C = carbon. *Denotes significant differences from the respective control treatments (*p<0.05, **p<0.01, ***p<0.001).”

#### 3.1.2 Nutrient levels in the model communities

No differences in levels of nitrite+nitrate (NO_2_^−^+NO_3_^−^, PERMANOVA: F_2,12_ = 0.68, p = 0.52) were found between the treatments. Although the main test indicated differences in ammonium (NH_4_^+^) levels among the treatments (PERMANOVA: F_2,12_ = 4.3, p = 0.034) only close to significantly higher levels pf ammonium were found in P1000, compared to the control (pair-wise PERMANOVA: p = 0.054) and to P100 (pair-wise PERMANOVA: p = 0.061, Table 1). A similar pattern was found for the total amount of nitrogen (Tot-N, PERMANOVA: F_2,12_ = 13.8, p = 0.003), where the concentration in P1000 was higher than in both the control (69% higher, pair-wise PERMANOVA: p = 0.0026) and P100 (56% higher, pair-wise PERMANOVA: p = 0.0067, Table 1).

The concentration of nitrite + nitrate was lower in all treatments compared to seawater blanks (PERMANOVA: F_3,14_ = 8.2, p<0.003; pair-wise PERMANOVA: p<0.05 for all treatments) while the levels of ammonium (PERMANOVA: F_3,14_ = 17, p<0.001; pair-wise PERMANOVA:, p<0.01 for all treatments) and total nitrogen (PERMANOVA: F_3,14_ = 19, p<0.001; pair-wise PERMANOVA: p<0.01 for all treatments) were higher in all treatments compared to seawater blanks.

The concentrations of phosphate (PO_4_^3−^) were several orders of magnitude higher in all experimental aquaria compared to seawater blanks, due to the continuous addition of the phosphoric acid buffer that propranolol was dissolved in (also added to the controls). No difference in phosphate was found between the treatments (PERMANOVA: F_2,12_ = 1.54, p = 0.25, Table 1).

### 3.2 Effects on the Separate Community Components

#### 3.2.1 Responses of the macroalga: Gross Production (GP), respiration (R) and GP∶R-ratio

The macroalga GP∶R-ratio of P1000 was the lowest of the three treatments, at both measurement occasions (Table 1). No statistical differences in macroalga GP∶R were however found between the treatments after 5 weeks of exposure (PERMANOVA: F_2,12_ = 0.72, p = 0.51, Table 1), nor after 6 weeks (PERMANOVA: F_2,12_ = 0.83, p = 0.47, Table 1), possibly due to the large within mean variation. The lower GP∶R in P1000 was initially the result of mainly low GP (5 weeks), and in the end a combination of low GP and high R (6 weeks). After 6 weeks the GP∶R displayed a non-linear pattern as the decrease GP∶R in P1000 remained (22% lower than the control), while the GP∶R in P100 was slightly increased (40% higher than the control), indicating what could be a hormesis effect.

#### 3.2.2 Responses of the macroalga: weight loss and carbon content

The macroalga in all replicates lost weight during the exposure period (Table 1). The weight loss seemed smaller in P1000 compared to both the control and P100, although there was no significant difference between the treatments (PERMANOVA: F_2,12_ = 2.4, p = 0.13). There were differences in macroalgae carbon content among the treatments (PERMANOVA: F_2,7_ = 22.4, p = 0.0015), where P1000 had higher carbon content than both the control (pair-wise PERMANOVA: p = 0.0004) and P100 (pair-wise PERMANOVA: p = 0.029, Table 1). P100 had slightly higher carbon content than the control (pair-wise PERMANOVA: p = 0.057).

#### 3.2.3 Responses of the mussel: respiration and feeding rate

The mussels’ respiration increased slightly by increasing propranolol exposure, and there were only close to significant differences among the treatments (PERMANOVA: F_2,12_ = 3.68, p = 0.060, Table 1).

There was also a trend of higher filtration rate of mussels in exposed treatments compared to the control, but there were large variances in filtration rate within all treatments and no significant differences were found (PERMANOVA: F_2,11_ = 0.43, p = 0.65, Table 1).

#### 3.2.4 Responses of the mussel: weight at end

The average mussel weights of both dead and surviving mussels at the end of the experiment were similar among all treatments (PERMANOVA: F_2,12_ = 1.7, p = 0.23), and neither were there any differences in mussel carbon content between the treatments (PERMANOVA: F_2,12_ = 1.24, p = 0.31, Table 1).

#### 3.2.5 Responses of the amphipod: respiration

The amphipods’ respiration was different between the treatments after the exposure (PERMANOVA: F_2,12_ = 3.9, p = 0.05). Amphipods exposed to P100 had 39% larger mean respiration than individuals from P1000 (pair-wise PERMANOVA: p = 0.041) and slightly, but not significantly, larger respiration than the control (pair-wise PERMANOVA: p = 0.073, Table 1).

#### 3.2.6 Responses of the amphipod: weight at end

There were no statistical differences between the weights of surviving amphipods at the end of the exposure (PERMANOVA: F_2,12_ = 0.76, p = 0.50), and neither were there any differences in amphipod carbon content between the treatments (PERMANOVA: F_2,9_ = 0.19, p = 0.89, Table 1).

#### 3.2.7 Responses of the amphipod: reproduction (number of juveniles)

At the end of the experiment there were amphipod juveniles in 11 of the 15 experimental aquaria. Despite a large variation in number of juveniles within each treatment, there were also differences between the treatments (glm: p<0.001) and there was a pattern of increasing number of juveniles with increasing concentration of propranolol. The number of juveniles in P1000 was significantly higher than in both the control (180% higher than control, Tukey: p<0.001, Table 1), and in P100 (49% higher than P100, Tukey: p<0.001). The number of juveniles was correlated to the number of surviving adults, with a slightly higher number of juveniles per adult in P1000, however, there was no significant difference between the treatments (PERMANOVA: F_2,12_ = 0.54, p = 0.61, Table 1).

#### 3.2.8 Responses of the sediment: GP∶R and carbon content

No statistical differences could be found in sediment GP∶R (PERMANOVA: F_2,12_ = 0.012, p = 0.99), among the treatments, although the results suggested a lower GP∶R for the treatments with propranolol, compared to the control (Table 1). Neither were there any differences in sediment carbon content between the treatments (PERMANOVA: F_2,12_ = 0.21, p = 0.81, Table 1).

#### 3.2.9 Mortality

**Figure 1:**
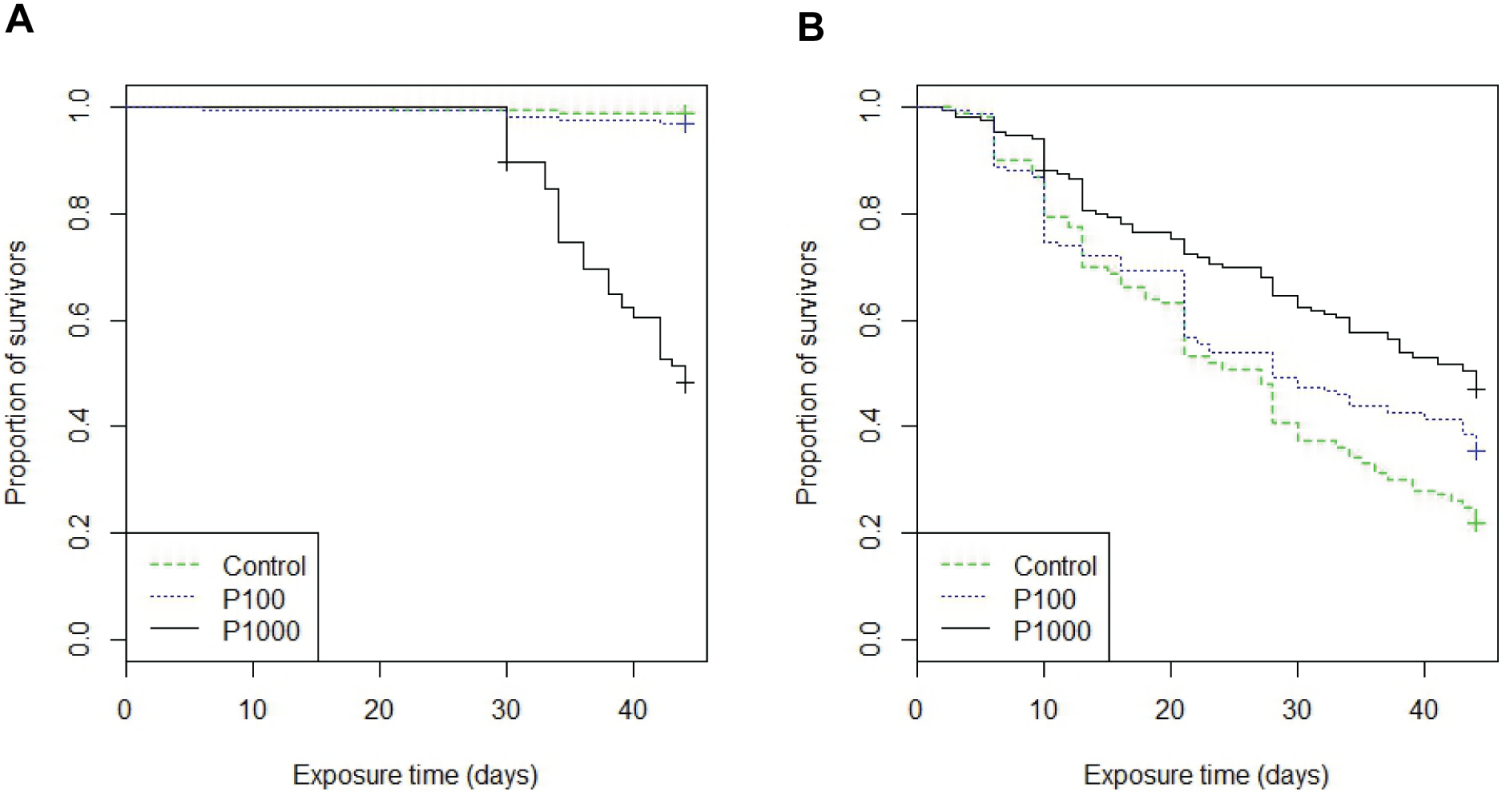
Mussel and amphipod mortality. Kaplan Meier survival curves depicting proportion of surviving individuals: A) mussels (*Mytilus edulis trossulus*), and B) amphipods (*Gammarus* spp.), after 6 weeks of exposure to propranolol in 0 μg l^−1^ (Control); 100 μg l^−1^ (P100) and 1000 μg l^−1^ (P1000).

The mussel mortality in general was relatively low but with large variations (overall mean 12.5%±9.5%). There were however differences between the treatments (glm: p<0.001) and the mortality increased with increasing concentration of propranolol (Table 1, Figure 1A). The mortality of P1000 was significantly higher than both the control (Tukey: p<0.001) and P100 (Tukey: p<0.001). There were differences also in amphipod mortality between the treatments (glm: p = 0.024), although amphipod mortality conversely was generally high (overall mean 68.4%±4%). An unexpected decrease in mortality with increasing concentration of propranolol was detected, where the mortality of P1000 was lower than of the control (Tukey: p = 0.019, Table 1, Figure 1B). At the end, a relatively large number of amphipods were missing (in total 27%), which was most probably due to cannibalism, as the amphipods were observed to feed on each other. The missing individuals were assumed to have been predated with an even distribution in time over the course of the experiment, and were included in such a manner in the Kaplan Meier amphipod survival curve (1B). The main mussel mortality of P1000 started after 30 days of exposure and continued to the end of the experiment, while the amphipod mortality was evenly distributed over the exposure period.

### 3.3 Propranolol in Water, Biota and Sediment

Propranolol was detected in all analysed components of P100 and P1000 at the end of the experiment (Table 2). There was an even concentration of propranolol in exposed aquaria over the length of the experiment and the measured concentrations were close to the nominal concentrations (8.2% deviation in P100 and 5.8% deviation in P1000). The highest concentrations and BCF was found in mussels from P1000, but also mussels from P100 and amphipods from both concentrations contained significant concentrations of propranolol. Most of the added propranolol was present in the water (80% in P100 and 77% in P1000), only 14% (P100) and 19% (P1000) in biota, and 6% and 4% respectively, in the sediment. For the sediment, amphipods and algae, the relative uptake declined with increased exposure, whereas for the mussels it was the other way around and the amount of propranolol was 60% higher in P1000, compared to P100.

**Table 2:** Distribution of propranolol in water, biota and sediment.

		Water	Mussels	Amphipods	Sediment	Algae
**Control**	Concentration	<LOQ	<LOQ	<LOQ	<LOQ	-
**P100**	Concentration	108±5.8	5.3±0.63	3.2±n.a.	0.61±0.047	-
	BCF or CF		46±6.7	70±n.a.	5.6±0.52	-
	Distribution	80%	11%	0.30%	6%	2%
**P1000**	Concentration	1058±37	89±11	6.3±n.a.	4.0±0.043	-
	BCF or CF		87±11	45±n.a.	3.9±0.37	-
	Distribution	77%	18%	0.10%	4%	0.50%

Concentration of propranolol in water (μg l^−1^), biota (μg g ww^−1^) and sediment (μg g ww^−1^). Bioconcentration factor (BCF: (mg kg ww^−1^)/(mg l^−1^))) determined for mussels and amphipods and concentration factor (CF: (mg kg ww^−1^)/(mg l^−1^)) for sediment. Propranolol distribution (%) among the components in the microcosms (assuming a similar concentration in the algae as in the amphipods). Quantifications made after exposure to propranolol in 0 (Control); 100 μg l^−1^ (P100) and 1000 μg l^−1^ (P1000), mean±se. One mussel replicate from P1000 (one individual) showed a considerably higher concentration of propranolol (310 μg g ww^−1^) and BCF (295) than the other analysed individuals from the same treatment, and was excluded from the overall mean. LOQ = level of quantification, ww = wet weight.

### 3.4 Visual Observations

There were clear differences in colour of the macroalga and of bacteria/epiphytes on the aquaria walls, between the treatments. The macroalga turned from red to different shades of red/brown/green in all but one treatment from P100, evenly distributed among the treatments. The aquaria walls of the control and P100 replicates were covered in brown biofilm at the end of the exposure. There was however almost none of this biofilm in P1000, which instead had a much higher ratio of red and green colonies, presumably different bacterial colonies, on the aquaria walls, compared to the controls. Black colonies were found on the aquaria walls in one of the P1000 replicates.

### 3.5 Interactions among the Separate Components

**Figure 2:**
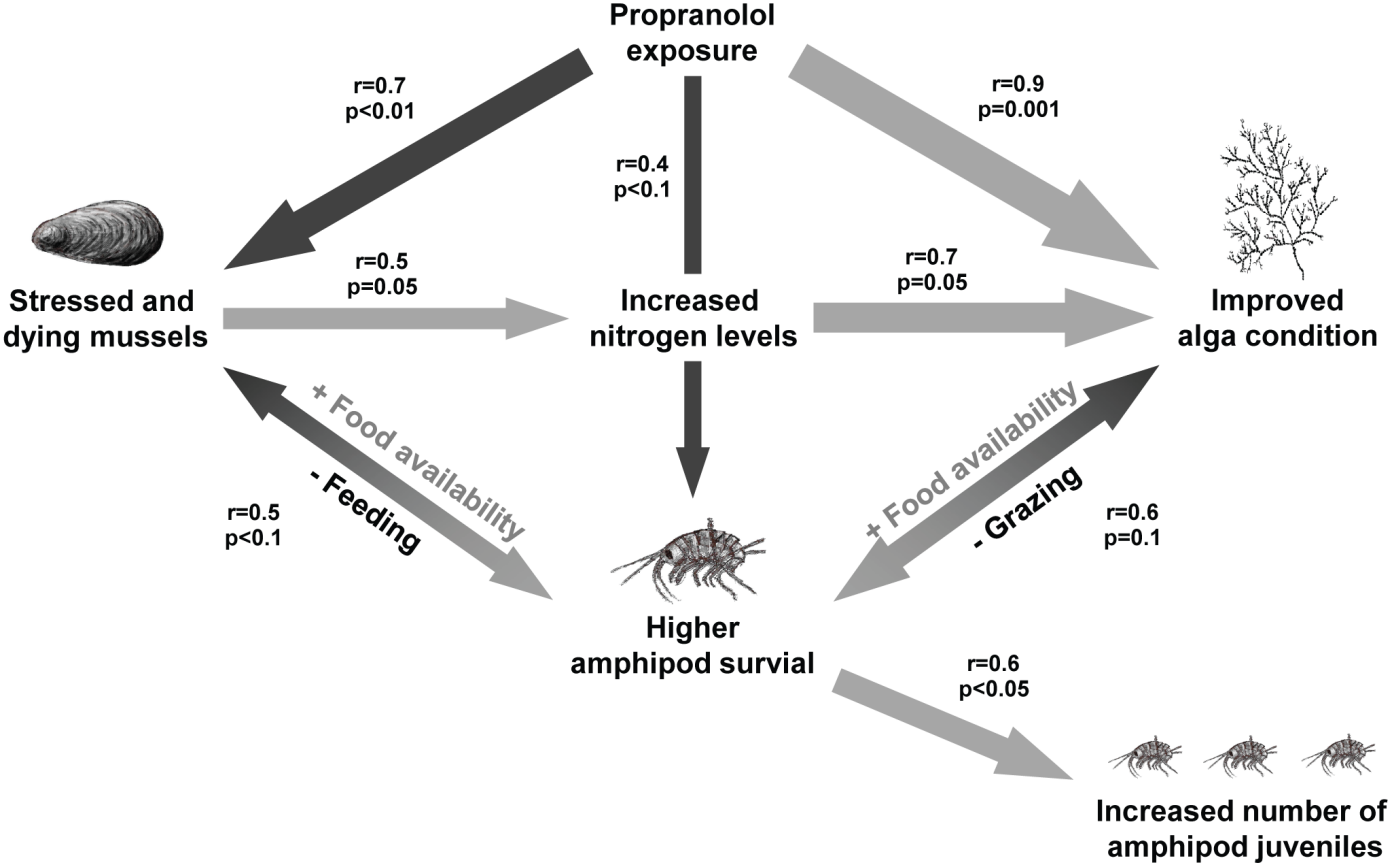
Conceptual model of interactions and indirect effects within the model communities. The interactions are determined by correlation coefficients. The width of the arrow denotes the strength of the correlation. Black arrows illustrate a negative influence on the organism whereas grey arrows illustrate a positive influence.

Based on the detected effects on the separate components and especially the unexpected decrease in amphipod mortality with increasing exposure, as well as the smaller weight loss and increased carbon content in exposed macroalgae, a conceptual model of relationships among the species and possible indirect effects of the exposure was formed (Figure 2). Correlation analyses confirmed several of the conceived links.

The number of dead mussels correlated with the increased total nitrogen concentration in the water (Pearson: r = 0.52, p = 0.05) and the increased nitrogen levels further correlated well with increased macroalgae carbon content (Pearson: r = 0.69, p = 0.028). Lower amphipod mortality correlated well with increased number of amphipod offspring (Pearson: r = 0.59, p = 0.02). Amphipod mortality was found to be nearly significantly correlated to both macroalgae carbon content (Pearson: r = 0.56, p = 0.091) and mussel mortality (Pearson: r = 0.48, p = 0.078).

## Discussion

Exposure to propranolol in this study resulted in several typical stress responses in mussels (increased mortality, increased respiration and increased excretion) [45], possible hormesis responses (macroalga GP∶R, amphipod respiration, sediment carbon content and community nitrite+nitrate concentrations) [31], [46], as well as some unexpected indirect positive effects (decreased amphipod mortality, decreased macroalga weight loss and increased macroalga carbon content). The hypothesis that buffering processes would reduce the effects of propranolol in exposed organisms compared to similar doses in single species experiments could, however, only partly be corroborated. The mussels were affected to a similar extent as in the single species experiments [31], while the additional organisms were less, or even positively, affected by the propranolol exposure: the macroalga with regard to biomass and carbon content; and the amphipods with regard to biomass and mortality. In previous single-species experiments, propranolol both reduced chlorophyll fluorescence and GP∶R in a macroalga (*Fucus vesiculosus*) [33], and the activity and physiology of amphipods [32], [33]. In this multi-species experiment, the toxic effects of propranolol were thus masked or reduced by ongoing interactions, and inter-specific relations between the different components buffered for effects of propranolol previously detected in less complex experimental systems with similar settings [31]–[33]. We therefore conclude that this is an example of positive indirect effects within communities that reduce or counter direct negative effects of contaminants and other stressors through inter-specific species interactions [47]–[49].

When scrutinising the conceptual model in detail (Figure 2), there were several interactions and processes responsible for the observed responses, and sometimes lack of expected negative effects. The starting point was the significant relationship between mussel mortality and pharmaceutical concentration. The poor condition of mussels, regarding the mortality, induced a series of interactions in the microcosms. The mussel degradation increased the nitrogen concentrations in P1000, mainly ammonium, which is a strong indication of degradation of organic matter [50]. The excretion from stressed animals likely made a minor contribution [45]. It was repeatedly observed how amphipods fed on dying mussels, which did not seem to have their normal ability to close their shells in protection. The substantial feeding of amphipods on mussels hence increased the degradation and release of nutrients into the water, at the same time as the amphipods shifted from feeding on the macroalga to the mussel. Less grazing from amphipods, together with increased levels of nutrients in the water, favoured the macroalga, despite the direct negative effects on the alga from the propranolol exposure. Ammonium is furthermore more bioavailable to macroalga than nitrite+nitrate [51], which is also reflected by the good condition of the macroalga in the treatments where the ammonium levels were high, that is, P1000. The amphipods in all treatments suffered from a generally high mortality, which could have been caused by several reasons, like natural death due to the end of life cycles, which for some of the sampled amphipods probably occurred during the exposure period [52], [53], the laboratory conditions, or cannibalism [54]. For the amphipods in the P1000 systems, the dying mussels, however, provided higher availability and better quality of food. This in turn caused higher amphipod weight, lower mortality and slightly increased carbon content and number of offspring, which was corroborated with significant correlations. If considering the observed negative effects of propranolol on macroalga and amphipods in previous studies [32], [33], it is reasonable to conclude that the positive effects of propranolol on these organisms were beneficial indirect effects induced by increased mussel mortality. Such interactions make an ecosystem susceptible for this type of unforeseen trophic cascade effect [2], [55].

There were no confounding effects due to buffer addition or nutrient availability in the experimental systems. The high phosphate concentrations in all aquaria were due to the daily addition of the solvent buffer, which contained phosphoric acid. No aquaria suffered from N-limitation, despite the increased phosphorous levels, as the nitrogen levels were higher in all treatments than in the seawater blanks.

High biodiversity of a community or an ecosystem is considered to provide increased stability, functioning and recovery potential [56], which correlate both to its response to disturbances [1], [3] and to its ecological resilience [57]. Communities with low diversity and resilience would hence generally be more sensitive to stressors. Contaminants will thus have a more severe effect in a low-biodiversity ecosystem, like the Baltic Sea, where there is a limited possibility for other organisms to regulate the same ecosystem function, and where the loss or change in function could affect the entire ecosystem [58], [59]. The mussel used in this experiment has such a fundamental function in the Baltic Sea, as it is a significant benthic filter feeder. The extensive beds they form exert significant effects on benthic-pelagic coupling and energy flows through their filtration activities [60], [61], which influence the structure and abundance of the associated community [62]. The high mussel mortality in this community exposure scenario is likely an effect from their substantial uptake of propranolol. The concentrations in mussel tissues were up to an order of magnitude higher than in the amphipod and sediment, and although they only contributed to 0.3% of the total mass in the experimental system, nearly 20% of the propranolol ended up in the mussels. The uptake also increased with increasing exposure, both in terms of tissue concentration, bioconcentration and relative amount in the system. The high uptake and sensitivity of the mussel is likely due to its filter feeding behaviour. *In situ*, the combination of contaminant exposure and a long life cycle, render a chronic exposure that makes the mussels especially vulnerable.

From a community ecology perspective, contaminant induced mortality has similarities with predation [63], [64]. In this case propranolol acted as a predator on the mussels. It has been shown that removal of a competitively dominant species by contaminants induces positive responses in inferior organisms, reviewed by [64]. The blue mussels of the Baltic Sea are important ecosystem engineers and facilitators of high biodiversity by creating both substrate and other livelihood prerequisites for associated species [65], [66], and by improving the water quality [61]. In spite of this, their mortality in the microcosm experiment induced positive indirect effects on the alga and amphipod, although such effects are likely smaller in a real Baltic Sea exposure scenario. Previous studies [32], [33] have demonstrated negative effects of pharmaceutical exposure also on macroalga. Eutrophication of the Baltic Sea results in increased algae growth, and a subsequent low grazing pressure; factors that this study have shown to counter the toxic effects of propranolol to macroalga, at least temporarily. If the observed positive indirect effects on the amphipod would remain in a real exposure scenario, is more difficult to predict. In a eutrophied environment there will always be high food availability for grazers; one variable that contributed to the positive indirect effects on the amphipods in this microcosm study. However, as amphipods are omnivorous organisms, it might as well be the quality of the food, that is, the higher energy content in the dying mussels compared to the algae, that was the decisive factor providing the prerequisites needed to sustain the stress caused by the propranolol, which was detected in earlier single-species studies [32], [33]. Thus, if the coastal area of the Baltic Sea would be exposed to pharmaceuticals to the extent that for example mussels would be severely affected, the exposure would likely not initially exert any negative effects on the amphipods. However, in a longer perspective of the low-biodiversity Baltic Sea, functional changes in the blue mussel population would affect the whole coastal zone. In addition, the detected amounts of propranolol in the sediment imply that also benthic organisms are at risk for exposure if pharmaceuticals reach the recipient in larger quantities. Although the potential effects of pharmaceuticals on sediment organisms are yet unknown, the results of this and previous single species studies [31]–[33] show that pharmaceutical exposure may have implications on the coastal ecosystem of the Baltic Sea.

## Conclusions

The study of effects in microcosms demonstrates a feasible yet relevant way of observing possible environmental effects. This multi-species exposure experiment revealed uptake and effects of the human pharmaceutical propranolol on non-target organisms. The most sensitive species, the mussel, was affected to the same extent as in previous single species studies [31]–[33], while the effects on amphipods and algae were smaller, or even positive, compared to in less complex test systems. This was likely due to compensatory effects within the model communities, as a result of both negative and positive interactions. Buffering processes resulting in smaller net effects have been observed also in experiments with other variables [47] and contaminant exposure can even induce more indirect than direct effects [63]. The combination of effects of both direct and indirect character would not have been detected if using simpler experimental set-up, with fewer community components. If a more complex experimental set-up is used, buffering effects between organisms having the same or a similar biological role may on the other hand reduce the possibilities of detecting interaction effects. It is, however, the combination of effects with interactions among species that will be found in the environment. This exposure study hence contributes to the understanding of the direct effects of pharmaceuticals in the environment as well as their possible subsequent effects on both community function and structure. The similar response patterns between the treatments indicate that also lower concentrations may affect a community in the field, especially in a more chronic exposure scenario. The structural changes found, in terms of survival and biomass of the mussels, macroalga and amphipods, and especially the increased amphipod propagation, would in the natural environment have a long-term influence on the ecosystem function.

## References

[1] Hughes AR (2012) Disturbance and Diversity: An Ecological Chicken and Egg Problem. Nature Education Knowledge 3: 48.

[2] Fleeger J, Carman K, Nisbet R (2003) Indirect effects of contaminants in aquatic ecosystems. Science of the Total Environment 317: 207–233. 14630423 10.1016/S0048-9697(03)00141-4

[3] Hooper D, Chapin F III, Ewel J, Hector A, Inchausti P, et al (2005) Effects of biodiversity on ecosystem functioning: a consensus of current knowledge. Ecological Monographs 75: 3–35.

[4] Fent K, Weston A, Caminada D (2006) Ecotoxicology of human pharmaceuticals. Aquatic Toxicology 76: 122–159. 16257063 10.1016/j.aquatox.2005.09.009

[5] Forbes V, Calow P (2012) Promises and problems for the new paradigm for risk assessment and an alternative approach involving predictive systems models. Environmental Toxicology and Chemistry 31: 2663–2671. 23165997 10.1002/etc.2009

[6] Sheehan PJ (1984) Effects on community and ecosystem structure and dynamics. In: PJ S, Miller D, GC B, Ph B, editors. Effects of pollutants at the ecosystem level SCOPE 22. New York, USA: John Wiley & Sons Ltd. 51–100.

[7] Forbes V, Calow P (1999) Is the per capita rate of increase a good measure of population-level effects in ecotoxicology? Environmental Toxicology and Chemistry 18: 1544–1556.

[8] Holling C (1973) Resilience and stability of ecological systems. Annual Review of Ecology and Systematics 4: 1–23.

[9] Rönnbäck P, Kautsky N, Pihl L, Troell M, Söderqvist T, et al (2007) Ecosystem Goods and Services from Swedish Coastal Habitats: Identification, Valuation, and Implications of Ecosystem Shifts. Ambio 36: 534–544. 18074889 10.1579/0044-7447(2007)36[534:egasfs]2.0.co;2

[10] Elmgren R (2001) Understanding human impact on the Baltic ecosystem: Changing views in recent decades. Ambio 30: 222–231. 11697254

[11] Wulff F, Bonsdorff E, Gren IM, Johansson S, Stigebrandt A (2001) Giving advice on cost effective measures for a cleaner Baltic Sea: A challenge for science. Ambio 30: 254–259. 11697258

[12] Jansson B-O (1980) Natural Systems of the Baltic Sea. Ambio 9: 128–136.

[13] Elmgren R, Hill C (1997) Ecosystem function at low biodiversity - The Baltic example. In: Ormond RFG, Gage J and Angel M, editors. Marine Biodiversity: Patterns and processes, Cambridge University Press. 319–336.

[14] Kautsky U (1995) Ecosystem processes in coastal areas of the Baltic Sea. Doctoral thesis, Stockholm University, Stockholm, Sweden.

[15] Norling P (2009) Importance of blue mussels for biodiversity and ecosystem functioning in subtidal habitats. Doctoral thesis, Stockholm University, Stockholm, Sweden.

[16] Jjemba P (2006) Excretion and ecotoxicity of pharmaceutical and personal care products in the environment. Ecotoxicology and Environmental Safety 63: 113–130. 16399163 10.1016/j.ecoenv.2004.11.011

[17] Bengtsson B-E, Gunnarsson B, Wall T, Wennmalm Å, editors (2005) Läkemedel och miljö. Apoteket AB. 148 p.

[18] Bendz D, Paxéus NA, Ginn TR, Loge FJ (2005) Occurrence and fate of pharmaceutically active compounds in the environment, a case study: Hoje River in Sweden. Journal of Hazardous Materials 122: 195–204. 15967274 10.1016/j.jhazmat.2005.03.012

[19] Wahlberg C, Björlenius B, Paxéus N (2010) Läkemedelsrester i Stockholms vattenmiljö. Förekomst, förebyggande åtgärder och rening av avloppsvatten. Stockholm Vatten AB. 141 p.

[20] Andreozzi R, Raffaele M, Nicklas P (2003) Pharmaceuticals in STP effluents and their solar photodegradation in aquatic environment. Chemospere 50: 1319–1330. 10.1016/s0045-6535(02)00769-5 12586163

[21] Falås P, Andersen HR, Ledin A, la Cour Jansen J (2012) Occurence and reduction of pharmaceuticals in the water phase at Swedish wastewater treatment plats. Water Science and Technology 66: 783–791. 22766867 10.2166/wst.2012.243

[22] Alexander BS, Wood MD (1987) Stereoselective Blockade of Central [H-3] 5-Hydroxytryptamine Binding to Multiple Sites (5-Ht1a, 5-Ht1b and 5-Ht1c) by Mianserin and Propranolol. Journal of Pharmacy and Pharmacology 39: 664–666. 2888864 10.1111/j.2042-7158.1987.tb03452.x

[23] Escher BI, Bramaz N, Richter M, Lienert J (2006) Comparative ecotoxicological hazard assessment of beta-blockers and their human metabolites using a mode-of-action-based test battery and a QSAR approach. Environmental Science & Technology 40: 7402–7408. 17180995 10.1021/es052572v

[24] Huggett D, Brooks B, Peterson B, Foran C, Schlenk D (2002) Toxicity of select beta adrenergic receptor-blocking pharmaceuticals (B-blockers) on aquatic organisms. Archives of Environmental Contamination and Toxicology 43: 229–235. 12115049 10.1007/s00244-002-1182-7

[25] Adamo SA (2008) Norepinephrine and octopamine: linking stress and immune function across phyla. Invertebrate Survival Journal 5: 12–19.

[26] Lacoste A, Malham SK, Cueff A, Poulet SA (2001) Noradrenaline modulates oyster hemocyte phagocytosis via a beta-adrenergic receptor-cAMP signaling pathway. General and Comparative Endocrinology 122: 252–259. 11356037 10.1006/gcen.2001.7643

[27] Massarsky A, Trudeau VL, Moon TW (2011) beta-Blockers as Endocrine Disruptors: The Potential Effects of Human beta-Blockers on Aquatic Organisms. Journal of Experimental Zoology Part a-Ecological Genetics and Physiology 315A: 251–265. 10.1002/jez.672 21370487

[28] Cleuvers M (2003) Aquatic ecotoxicity of pharmaceuticals including the assessment of combination effects. Toxicology Letters 142: 185–194. 12691712 10.1016/s0378-4274(03)00068-7

[29] Ferrari B, Mons R, Vollat B, Fraysse B, Paxeus N, et al (2004) Environmental risk assessment of six human pharmaceuticals: Are the current environmental risk assessment procedures sufficient for the protection of the aquatic environment? Environmental Toxicology and Chemistry 23: 1344–1354. 15180389 10.1897/03-246

[30] Stanley JK, Ramirez AJ, Mottaleb M, Chambliss CK, Brooks BW (2006) Enantiospecific toxicity of the beta-blocker propranolol to Daphnia magna and Pimephales promelas. Environmental Toxicology and Chemistry 25: 1780–1786. 16833138 10.1897/05-298r1.1

[31] Ericson H, Thorsén G, Kumblad L (2010) Physiological effects of diclofenac, ibuprofen and propranolol on Baltic Sea blue mussels. Aquatic Toxicology 99: 223–231. 20554059 10.1016/j.aquatox.2010.04.017

[32] Eriksson Wiklund A-K, Oskarsson H, Thorsén G, Kumblad L (2011) Behavioural and physiological responses to pharmaceutical exposure in macroalgae and grazers from a Baltic Sea littoral community. Aquatic Biology 14: 29–39.

[33] Oskarsson H, Eriksson Wiklund A-K, Lindh K, Kumblad L (2012) Effect studies of human pharmaceuticals on *Fucus vesiculosus* and *Gammarus* spp. Marine Environmental Research 74: 1–8. 22189068 10.1016/j.marenvres.2011.11.001

[34] Ankar S, Elmgren R (1978) The benthic macro- and meiofauna of the Askö-Landsort area (northern Baltic proper). A stratified random sampling survey. Contributions from the Askö Laboratory, Stockholm University, Sweden 11: 115 p.

[35] Kautsky H (1989) Quantitative distribution of plant and animal communities of the phytobenthic zone in the Baltic Sea. Contributions from the Askö Laboratory, Stockholm University, Sweden 35: 80 p.

[36] Jansson B-O, Wulff F (1977) Ecosystem analysis of a shallow sound in the northern Baltic – a joint study by the Askö group. Contributions from the Askö Laboratory, Stockholm University, Sweden 18: 160 p.

[37] Lora-Vilchis MC, Cordero-Esquivel B, Voltolina D (2004) Growth of *Artemia franciscana* fed *Isochrysis* sp. and *Chaetoceros muelleri* during its early life stages. Aquaculture Research 35: 1086–1091.

[38] Zhu CJ, Lee YK (1997) Determination of biomass dry weight of marine microalgae. Journal of Applied Phycology 9: 189–194.

[39] Clausen I, Riisgard HU (1996) Growth, filtration and respiration in the mussel *Mytilus edulis*: No evidence for physiological regulation of the filter-pump to nutritional needs. Marine Ecology Progress Series 141: 37–45.

[40] Maire O, Amouroux J-M, Duchêne J-C, Grémare A (2007) Relationship between filtration activity and food availability in the Mediterranean mussel *Mytilus galloprovincialis* . Marine Biology 152: 1293–1307.

[41] Prevodnik A, Gardeström J, Lilja K, Elfwing T, McDonagh B, et al (2007) Oxidative stress in response to xenobiotics in the blue mussel *Mytilus edulis* L.: Evidence for variation along a natural salinity gradient of the Baltic Sea. Aquatic Toxicology 82: 63–71. 17320983 10.1016/j.aquatox.2007.01.006

[42] Ericson H, Thorsén G, Kumblad L (2010) Physiological effects of diclofenac, ibuprofen and propranolol on Baltic Sea blue mussels. Aquatic Toxicology 99: 223–231. 20554059 10.1016/j.aquatox.2010.04.017

[43] Anderson MJ (2001) A new method for non-parametric multivariate analysis of variance. Austral Ecology 26: 32–46.

[44] McArdle BH, Anderson MJ (2001) Fitting multivariate models to community data: A comment on distance-based redundancy analysis. Ecology 82: 290–297.

[45] Widdows J (1985) Physiological measurements. In: Bayne B, Brown D, Burns K, Dixon D, Ivanovici A et al., editors. The effects of stress and pollution and marine animals. New York: Praeger. 3–45.

[46] Calabrese EJ (2008) Hormesis: Why it is important to toxicology and toxicologists. Environmental Toxicology and Chemistry 27: 1451–1474. 18275256 10.1897/07-541

[47] Alsterberg C, Eklöf JS, Gamfeldt L, Havenhand JN, Sundbäck K (2013) Consumers mediate the effects of experimental ocean acidification and warming on primary producers. PNAS 110: 8603–8608. 23630263 10.1073/pnas.1303797110 PMC3666745

[48] Shure DJ (1971) Insecticide effects on early succession in an old ecosystem. Ecology 52: 271–279.

[49] Relyea R, Schoeppner N, Hoverman J (2005) Pesticides and amphibians: The importance of community context. Ecological Applications 15: 1125–1134.

[50] Smith RL, Smith TM (2003) Elements of Ecology. San Francisco: Benjamin Cummnings.

[51] Ruist E (2008) Fosfor- och kvävefraktioner i miljöövervakningen – En studie av bohuslänska vattendrag. Länsstyrelsen Västra Götaland län.

[52] Kolding S (1981) Habitat Selection and Life-Cycle Characteristics of 5 Species of the Amphipod Genus Gammarus in the Baltic. Oikos 37: 173–178.

[53] Kolding S, Fenchel TM (1981) Patterns of Reproduction in Different Populations of 5 Species of the Amphipod Genus Gammarus. Oikos 37: 167–172.

[54] MacNeil C, Dick JTA, Elwood RW (1999) The dynamics of predation on Gammarus spp. (Crustacea : Amphipoda). Biological Reviews 74: 375–395.

[55] Relyea R, Diecks N (2008) An unforseen chain of events: lethal effects of pesticides on frogs at sublethal concentrations. Ecological Applications 18: 1728–1742. 18839767 10.1890/08-0454.1

[56] Worm B, Barbier E, Beaumont N, Duffy J, Folke C, et al (2006) Impacts of biodiversity loss on ocean ecosystem services. Science 314: 787–790. 17082450 10.1126/science.1132294

[57] Gunderson L (2000) Ecological resilience – in theory and application. Annual Review of Ecology and Systematics 31: 425–439.

[58] Janson B-O (1980) Natural systems of the Baltic Sea. Ambio 9: 128–136.

[59] Bonsdorff E, Pearson T (1999) Variation in the sublittoral macrozoobenthos of the Baltic Sea along environmental gradients: A functional-group approach. Australian Journal of Ecology 24: 312–326.

[60] Kautsky N, Evans S (1987) Role of Biodeposition by *Mytilus edulis* in the Circulation of Matter and Nutrients in a Baltic Coastal Ecosystem. Marine Ecology Progress Series 38: 201–212.

[61] Newell RIE (2004) Ecosystem influences of natural and cultivated populations of suspension-feeding bivalve molluscs: A review. Journal of Shellfish Research 23: 51–61.

[62] Norling P, Kautsky N (2007) Structural and functional effects of *Mytilus edulis* on diversity of associated species and ecosystem functioning. Marine Ecology Progress Series 351: 163–175.

[63] Clements WH, Rohr JR (2009) Community Responses to Contaminants: Using Basic Ecological Principles to Predict Ecotoxicological Effects. Environmental Toxicology and Chemistry 28: 1789–1800. 19358627 10.1897/09-140.1

[64] Rohr JR, Kerby JL, Sih A (2006) Community ecology as a framework for predicting contaminant effects. Trends in Ecology & Evolution 21: 606–613. 16843566 10.1016/j.tree.2006.07.002

[65] Norling P, Kautsky N (2008) Patches of the mussel *Mytilus* sp are islands of high biodiversity in subtidal sediment habitats in the Baltic Sea. Aquatic Biology 4: 75–87.

[66] Koivisto M, Westerbom M, Riihimaki A (2011) Succession-driven facilitation of macrofaunal communities in sublittoral blue mussel habitats. Marine Biology 158: 945–954.

